# Editorial: Gonadotropin-Releasing Hormone Receptor Signaling and Functions

**DOI:** 10.3389/fendo.2018.00143

**Published:** 2018-04-04

**Authors:** Ivana Bjelobaba, Stanko S. Stojilkovic, Zvi Naor

**Affiliations:** ^1^Department of Neurobiology, Institute for Biological Research “Sinisa Stankovic”, University of Belgrade, Belgrade, Serbia; ^2^National Institute of Child Health and Human Development, National Institute of Health, Bethesda, MD, United States; ^3^Department of Biochemistry and Molecular Biology, Tel Aviv University, Tel Aviv, Israel

**Keywords:** GnRH receptor, GnRH, pituitary gland, GPCR-signal transduction, gonadotropes

**Editorial on the Research Topic**

**Gonadotropin-Releasing Hormone Receptor Signaling and Functions**

The hypothalamic decapeptide gonadotropin-releasing hormone, termed GnRH or GnRH1, and its receptor expressed in pituitary gonadotrophs, termed GnRHR or GnRHR1, play a central role in vertebrate reproduction. In gonadotrophs, GnRHR activation leads to InsP_3_-dependent oscillatory calcium signaling and protein kinase C activation, accompanied with periodic changes in electrical activity and voltage-gated calcium influx. These receptors also trigger multiple lipid-derived messengers and mitogen-activated protein kinases activation, ultimately controlling transcription of numerous genes and gonadotropin secretion. Unlike other vertebrate GnRHRs and all other G-protein coupled receptors, mammalian GnRHRs lack a C-terminal tail, which makes them more resistant to desensitization and internalization. This peculiarity led to research of GnRHR trafficking and subsequent identification of mutations that are affecting human fertility. Also, the GnRH ligand–receptor system became an important target in assisted reproductive technologies in humans and domestic animals and in some cancers, prostate cancer in particular.

GnRHRs appear early in the evolution of invertebrates; their natural ligands are beginning to emerge, and their functions are not necessarily related to reproduction. Diverse forms of GnRH and GnRHR have also been identified in vertebrates, including GnRH2 and its receptor GnRHR2. The vertebrate GnRHR is also found in extrapituitary sites, including central nervous system, reproductive tissues, and cancer cells derived from such tissues. The enhanced interest for the extrapituitary GnRH ligand–receptors systems also comes from the findings that they mediate antiproliferative and/or proapoptotic effects and may, therefore, be directly targeted in cancer therapy. This collection of original research articles, reviews, perspectives, and hypotheses and theories summarizes well our current knowledge of GnRHR evolution, structure and regulation, mechanisms of pulsatile GnRH release, the roles of GnRH ligands and receptors in cellular functions, and practical application of this knowledge.

In vertebrates, activation of GnRHR in gonadotrophs and gonadotropin release relies on pulsatile GnRH secretion from the hypothalamus, but the mechanism underlying these pulses is still not well characterized. In her review, Constantin summarizes the current knowledge of the physiology of hypothalamic GnRH neurons in terms of excitability and secretion and the proposed mechanisms for synchronization of electrical activity to generate pulsatile GnRH release.

The crystal structure of GnRHR is not yet resolved, which limits our understanding of GnRH binding to its receptors in gonadotrophs and their subsequent activation. In their review article, Flanagan and Manilall discuss how the crystal structures of related GPCRs could help in clarification of GnRHR structure, ligand binding to the receptor ectodomain and transduction of signals to cytoplasmic domain and heterotrimeric G_q/11_ proteins.

Pituitary gonadotrophs are excitable cells and various voltage- and ligand-gated and related channels provide a background pathway for spontaneous firing of action potentials and calcium signaling. Activation of GnRHR and other calcium-mobilizing receptors in these cells leads to calcium release from endoplasmic reticulum through IP_3_R channels coupled with a rapid gonadotropin secretion. This is followed by switch in the pattern of firing of action potentials from tonic single spiking to periodic plateau bursting, the latter being essential for sustained calcium signaling and gonadotropin release. Overview of these mechanisms and ion channels in gonadotrophs is given by Stojilkovic et al.

Two articles in this issue deal with GnRHR-mediated cell shape remodeling. Edwards et al. nicely summarize the gonadotroph cell network and plasticity *in vivo*. GnRH engages the actin cytoskeleton to not only increase cell movement but also causes membrane remodeling events in the form of membrane ruffles, filopodia, and lamellipodia, in order for the cell to gain access to the vasculature. The authors stress that the mechanisms of actin polymerization and activation of mitogen-activated protein kinases are still elusive.

On the other hand, Rahamim-Ben Navi et al. show that GnRH-induced membrane bleb formation in immortalized LβT2 gonadotrophs and mouse primary pituitary cells depends on ERK1/2 signaling. GnRHR, c-Src, ERK1/2, FAK, paxillin, and tubulin appear to be accumulated in the blebs, which the authors consider as yet another form of gonadotroph cell plasticity and as a normal response of cells to GnRH. Furthermore, members of the signalosome, which was previously described by the authors, migrate to the blebs, which are apparently involved in cell migration.

Three articles provide details about GnRH-induced gene expression in gonadotrophs. The article by Janjic et al. describes basal and regulated GnRHR gene transcription in mammalian gonadotrophs, and the role of GnRH in regulated transcription. In rat and mouse, GnRH-induced transcription of genes relies primarily on the protein kinase C signaling pathway, with subsequent activation of mitogen-activated protein kinases. In contrast to pulsatile, continuous GnRH application shuts off regulated but not basal transcription, suggesting that different branches of this signaling pathway control transcription.

GnRH–GnRHR signaling pathways regulate the expression of various other genes and evidence implies that this signaling also influences the chromatin organization of target genes. Melamed et al. integrate the latest findings on GnRH-induced alterations in the chromatin of gonadotroph signature genes. The authors stress that further characterization of epigenome of gonadotropin genes may have implications in fertility drug development as well as in cancer biology.

Tackling the exact same question and using combination of a novel gel bead-in-emulsion drop-seq method and genome-wide chromatin accessibility state, Ruf-Zamojski et al. bring us a sneak peek into epigenetic and single-cell transcriptional landscapes of LβT2 gonadotrophs during GnRH stimulation. The authors provide useful data sets, and report a putative FSH beta gene enhancer identified as highly open chromatin. Interestingly, while great variability in basal and GnRH-induced gene expression of individual cells was observed, the authors report no influence of cell cycle stage on the gene response to GnRH.

Two articles give further guidance on GnRH/GnRHR signaling in pituitary gonadotrophs from the physiological point of view. Odle et al. discuss the role of leptin in reproduction and bring us two hypotheses derived from the experiments in which leptin receptor gene expression in gonadotrophs was manipulated. The authors propose that the cyclic changes in pituitary GnRHR expression create a mechanism by which these cells are activated only when environmental conditions are optimal. They further suggested that leptin’s role in the permissive regulation of the reproductive cycle depends on timed events that involve multiple interactive target cells in the hypothalamo–pituitary–gonadal axis.

Terasaka et al. argue that reactive oxygen species (ROS) were often overlooked when it comes to our understanding of GnRHR signaling in gonadotrophs. Indeed, ROS can interfere with different GnRHR-induced signaling events, including MAPK activation. Moreover, the authors show that the monounsaturated fatty acid oleate can induce mitochondrial ROS in LβT2 cells. The authors conclude that appreciating ROS signaling in gonadotrophs may give us insights into integration of stress signaling and the reproductive axis.

In contrast to GnRH/GnRHR, the roles of GnRH2/GnRHR2 remain largely unexplored; reflecting the absence of expression or function of this receptor in many mammals, including the most used experimental animals, rat and mouse. In their review article, Desaulniers et al. give an overview of GnRHR2 expression across the mammalian species, its structure and up-to-now revealed biological functions, which are diverse and not related to pituitary gonadotroph functions. The brain receptors may have a role in coordination of interactions between nutritional status and sexual behavior, whereas the gonadal receptors may contribute to the control of reproduction by stimulating steroidogenesis.

GnRH–GnRHR signaling pathway is also operative in other peripheral tissues. For example, in women GnRH influences gastrointestinal motility, while in rat prolonged GnRH treatment induces enteric neuron cell death. The review article by Ohlsson discusses the roles and potential mechanisms of GnRH action on the level of gastrointestinal tract. The author proposes further investigation for definitive confirmation of the presence of GnRHR(s) in the gut.

We now know more on GnRHR signaling in cancer cells and it is clear that signal transduction in different cancer cell lines does not include classical players of the GnRHR signaling cascade in gonadotrophs, as described in a review article by Gründker and Emons. Antiproliferative effects of GnRH and its analogs in cancer cells seem to rely on coupling of the receptor to G_i/o_ signaling pathway and activation of a phosphotyrosine phosphatase.

Finally, Sakai et al. review is focused on structure and biological functions of GnRH, adipokinetic hormone, corazonin, and their related peptides in invertebrates. While GnRH regulates some aspects of reproduction in mollusks, it is clear that all of these interrelated peptides have other biological roles in invertebrates. The authors advocate that further research in invertebrates, including sequence analyses in different species, will give us more information on evolutionary processes and biological significance.

## Author Contributions

IB wrote the initial draft. SS and ZN edited and corrected the text.

## Conflict of Interest Statement

The authors declare that the research was conducted in the absence of any commercial or financial relationships that could be construed as a potential conflict of interest.

